# Characterization of sexually transmitted infections, their pharmacological treatment, and recurrence in a Colombian population

**DOI:** 10.7705/biomedica.5872

**Published:** 2021-10-15

**Authors:** Luis Fernando Valladales-Restrepo, Juan Alberto Ospina-Cano, María José Londoño-Serna, Jorge Enrique Machado-Alba

**Affiliations:** 1 Grupo de Investigación en Farmacoepidemiología y Farmacovigilancia, Universidad Tecnológica de Pereira-Audifarma S.A, Pereira, Colombia Universidad Tecnológica de Pereira Universidad Tecnológica de Pereira Pereira Colombia; 2 Grupo de Investigación Biomedicina, Facultad de Medicina, Fundación Universitaria Autónoma de las Américas, Pereira, Colombia Fundación Universitaria Autónoma de las Américas Fundación Universitaria Autónoma de las Américas Pereira Colombia

**Keywords:** Sexually transmitted diseases, urethritis, recurrence, male, condoms, public health, enfermedades de transmisión sexual, uretritis, recurrencia, masculino, condones, salud pública

## Abstract

**Introduction:**

Sexually transmitted infections are a public health problem worldwide. Their inadequate antimicrobial management has been associated with a higher risk of recurrence.

**Objective:**

To characterize the main sexually transmitted infections, the adherence to clinical practice guidelines, and the factors associated with recurrence in Colombia.

**Materials and methods:**

We conducted an observational study to identify the main sexually transmitted infections, the sociodemographic variables, and the pharmacological management in a patient cohort from a population database of 6.5 million people affiliated with the Colombian health system. We made a multivariate analysis to identify the variables associated with recurrence.

**Results:**

We detected 3,158 patients with a mean age of 41.8 ± 14.5 years, of whom 63.1% were men. We found 4.030 episodes of sexually transmitted infections, predominantly urethral syndrome (27.5%). Only 13.6% of patients with urethral syndrome, ulcerative syndrome, or genital warts were managed in compliance with clinical practice guidelines and 20.6% were dispensed condoms; 16.7% of patients had recurrences and being male (OR=1.32; 95%CI 1.08-1.63), <30 years old (OR=1.72; 95%CI 1.40-2.13), being treated in municipalities other than capital cities (OR=1.43; 95%CI 1.06-1.94), and having received inadequate treatment for the first episode (OR=1.93; 95%CI 1.52-2.39) were associated with recurrence.

**Conclusions:**

The majority of patients with sexually transmitted infections were not treated in compliance with clinical practice guidelines and those who did not have adequate management had a higher risk of recurrence.

## Resumen

**Introducción.:**

Las infecciones de transmisión sexual constituyen actualmente un problema de salud pública en el mundo. Su inadecuado tratamiento antimicrobiano se ha relacionado con un mayor riesgo de recurrencias.

**Objetivo.:**

Caracterizar las principales infecciones de transmisión sexual, el cumplimiento de las guías de práctica clínica de Colombia y los factores asociados con las recurrencias.

**Materiales y métodos.:**

Se hizo un estudio observacional para determinar las principales infecciones de transmisión sexual, las variables sociodemográficas y el tratamiento farmacológico en una cohorte de pacientes registrados en una base de datos poblacional de 6,5 millones de personas afiliadas al Sistema de Salud de Colombia. Se hizo un análisis multivariado para establecer las variables asociadas con las recurrencias.

**Resultados.:**

Se detectaron 3.158 pacientes; su edad media era de 41,8 ±14,5 años y 63,1 % de ellos correspondía a hombres; se encontraron 4.030 episodios de infecciones de transmisión sexual con predominio del síndrome uretral (27,5 %). El 13,6 % de los pacientes con síndrome uretral, ulceroso o con verrugas genitales se manejó según las guías de práctica clínica. El 20,6 % tenía acceso a condones y el 16,7 % presentó recurrencias. Ser hombre (OR=1,32; IC_95%_ 1,08-1,63), tener <30 años (OR=1,72; IC_95%_ 1,40-2,13), ser tratado en municipios distintos a ciudades capitales (OR=1,43; IC_95%_ 1,06-1,94) y haber recibido un tratamiento inadecuado en el primer episodio (OR=1,93; IC_95%_ 1,52-2,39) fueron factores asociados con las recurrencias.

**Conclusiones.:**

La mayoría de los pacientes con infecciones de transmisión sexual no fueron tratados según las guías de práctica clínica y quienes no tuvieron un manejo adecuado presentaban mayor riesgo de recurrencias.

Sexually transmitted diseases are a variety of clinical syndromes and infections caused by pathogens transmitted during sexual intercourse ^1^. In recent years, many experts have suggested calling them sexually transmitted infections instead because the concept of disease involves a medical problem, usually with clear signs and symptoms, while sexually transmitted diseases also affect asymptomatic patients and, therefore, not all infections can be considered diseases ^2^^,^^3^.

Sexually transmitted infections are among the most common contagious conditions affecting individuals’ health and lives around the world ^4^^,^^5^ and they are currently considered a global public health problem ^5^. Sexually transmitted infections prevalence depends on socioeconomic conditions, cultural and moral beliefs, and diagnostic and therapeutic options ^3^. According to the World Health Organization (WHO), every day, more than a million people acquire a sexually transmitted infection, and it has been estimated that 376 million new sexually transmitted infections cases occurred in 2016, among them, trichomoniasis (156 million), chlamydia (127 million), gonorrhea (87 million), and syphilis (6.3 million). Besides, the number of people with genital herpes exceeds 500 million and approximately 240 million suffer from chronic hepatitis B ^6^. In 2016, the prevalence of chlamydia infection in Colombian men was 9.2% and 7.4% among women while syphilis prevalence was 1.2% overall while gonorrhea prevalence was 0.7% in women and 0.6% in men ^7^.

These infections can cause acute urogenital conditions such as urethritis, cervicitis, vaginitis, and ulceration in the genitals, among others, and some may involve the rectum and pharynx. Several etiological agents are associated with serious short- and long-term complications ^4^. Sexually transmitted infections can be caused by viruses, bacteria, and parasites; they include mainly eight infections, four of which can be cured, namely, syphilis, gonorrhea, chlamydia, and trichomoniasis while the other four are caused by viruses (hepatitis B, herpes simplex, HIV and human papillomavirus) and are incurable, although there are treatments to attenuate, control, or modify the symptoms or the disease ^6^^,^^8^.

The lack of treatment is also considered a public health problem because untreated infections can lead to harmful consequences for individuals’ health ^9^. In this sense, the Colombian Ministry of Health and Social Protection and the Administrative Department of Science, Technology, and Innovation (Colciencias) published in 2013 the clinical practice guidelines for the syndromic approach to the diagnosis and treatment of patients with sexually transmitted infections and other genital tract infections aimed at providing health professionals with evidence on therapy effectiveness and safety to reduce the variability found in clinical practice, among other objectives ^10^. However, few studies in the country have addressed this topic and the pharmacological management indicated for patients with sexually transmitted infections is unknown. Therefore, we sought to characterize the main sexually transmitted infections, the adherence of their treatment to clinical practice guidelines, and the factors associated with recurrence in a Colombian population.

## Materials and methods

### Study design

We conducted an observational, descriptive, and retrospective cohort study on the treatment schemes used in the management of some sexually transmitted infections and their recurrence. Data were obtained from a population database of drugs dispensed that gathers information from about 6.5 million individuals affiliated with the contributory regime of the Colombian Health System through five insurers known in the country as health-promoting companies corresponding to approximately 30.0% of the actively affiliated population in this regime in the country and 14.3% of the Colombian total population.

We selected patients of either sex aged 14 or older treated in outpatient units from January 1 to December 31, 2015. We excluded those who had had a sexually transmitted infection in 2013 or 2014. We followed the patients until June 30, 2019. Besides, we analyzed the International Classification of Diseases, 10^th^ revision (ICD-10) codes related to sexually transmitted infections and the drugs prescribed for their management.

The ICD-10 diagnostic codes used for patient identification were the following:

• Urethral discharge syndrome and/or cervicitis:

» Urethritis: N341, N342, N370

» Gonococcal infections: A540-A542, A546-A549

» Chlamydial infections: A560-A563, A568

» Trichomoniasis: A590, A598, A599

• Ulcerative syndrome:

» Syphilis: A510-A512, A539, N742

» Genital herpes: A600, A601, A609

» Soft chancre: A57X

» Lymphogranuloma venereum: A55X

» Granuloma inguinale: A58X

» Anogenital warts: A630

• Others:

» Hepatitis B: B160-B162, B169, B170, B180, B181

» Hepatitis C: B171, B182

» HIV: B200-B213, B217-B222, B227, B230-B232, B238, B24X, F024, R75X.

Based on the information on the use of drugs by the affiliated population, systematically recorded by the dispensing company Audifarma SA, we designed a database to collect the following groups of variables:

1. Sociodemographic: sex, age, city of dispensation.

2. Chronic comorbidities: we identified the main cardiovascular, endocrine, rheumatic, urological, kidney, psychiatric, neurological, digestive, respiratory, and neoplastic diseases from the reported ICD-10 diagnostic codes.

3. Drugs used in the management of some sexually transmitted infections. Management was considered adequate when the prescribed antibiotic, dose, and duration of treatment followed the Colombian clinical practice guidelines (10), as follows:

» Urethral discharge syndrome or cervicitis:

» Urethritis: ceftriaxone (500 mg IM single dose) and azithromycin (1 g orally single dose)

» Gonococcal infections: ceftriaxone (500 mg IM single dose) or spectinomycin (2 g IM single dose)

» Chlamydial infections: azithromycin (1 g orally single dose) or doxycycline (100 mg orally administered every 12 hours for 7 days)

» Trichomoniasis: tinidazole (2 g oral single dose) or metronidazole (2 g orally single dose)

» Ulcerative syndrome:

» Syphilis: benzathine penicillin G (2,400,000 IU single-dose IM) or doxycycline (100 mg orally administered every 12 hours for 14 days)

» Genital herpes: acyclovir (200 mg orally administered 5 times a day for 6-7 days) or valaciclovir (1 g orally administered every 12 hours for 7-10 days)

» Soft chancre: azithromycin (1 g orally single dose) or ceftriaxone (250 mg IM single dose) or erythromycin (500 mg orally administered every 8 hours for 7 days)

» Lymphogranuloma venereum or granuloma inguinale: azithromycin (1 g orally administered once a week for 3 weeks) or doxycycline (100 mg orally administered 2 times a day for 21 days)

» Anogenital warts (1): podophyllin or trichloroacetic acid

» HIV, hepatitis B and C pharmacological management was not taken into account.

4. Inadequate management: All patients who did not receive pharmacological management and those who received another type of antimicrobial therapy not recommended by the clinical practice guidelines ^1^^,^^10^.

5. Recurrence: Patients with two or more sexually transmitted infection episodes through the follow-up period were considered recurrent.

6. Dispensing of hormonal contraceptives (oral, injectable, or depot) and/ or condoms.

### Data analysis

The data were analyzed with the SPSS Statistics, version 26.0 program for Windows (IBM, USA). We made a descriptive analysis with frequencies and proportions for the qualitative variables and measures of central tendency and dispersion for the quantitative variables. The quantitative variables were compared using the Student’s t-test or variance analysis and the categorical variables using the χ^2^ test. The binary logistic regression models used the presence of two or more sexually transmitted infections episodes (recurrence) as a dependent variable and those variables significantly associated with recurrence in the bivariate analyses as covariates. We adopted p<0.05 as the statistical significance level.

### Bioethical considerations

The protocol was approved by the Bioethics Committee of the *Universidad Tecnológica de Pereira* under the category of risk-free research (approval code: 01-071019). We adhered to the ethical principles established by the Declaration of Helsinki and used no personal patient data.

## Results

We identified 3,158 patients with a sexually transmitted infections diagnosis distributed in 64 different cities or municipalities of whom 63.1% (n=1994) were men. The mean age was 41.8 ± 14.5 years (range:14.0-96.8 years) distributed in the following age groups: <30 years (n=760; 24.1%), 30-49 years (n=1566; 49.6%), 50-64 years (n=568; 18.0%), and >65 years (n=264; 8.4%); 90.4% (n=2,856) of the patients resided in capital cities.

During the selection period and the follow-up, we found 4,030 sexually transmitted infections episodes, predominantly urethral syndrome (n=1108; 27.5%) distributed as follows: urethritis (n=912; 22.6%), gonorrhea (n=136; 3.4%), chlamydia (n=32; 0.8%), and trichomoniasis (n=28; 0.7%) followed by ulcerative syndrome (n=1,105; 27.4%): syphilis (n=555; 13.8%), genital herpes (n=540; 13.4%), and chancre (n=10; 0.3%), as well as genital warts (n=939; 23.3%), HIV (n=808; 20.0%), and hepatitis B or C (n=70; 1.7%); 83.3% (n=2630/3158) of the patients had a single sexually transmitted infections episode while 16.7% (n=528) had two or more. At some point during followup, 20.6% (n=649) of the patients were dispensed condoms and 21.0% (n=245/1164) of the women, hormonal contraceptives, mostly oral ones (n=210/1164; 18.0%).

### Sexually transmitted infections pharmacological management

A 13.6% (428/3152) of the urethral syndrome, ulcerative syndrome, and genital warts episodes were adequately managed. Genital warts were the most frequently treated in compliance with the clinical practice guidelines (n=256/939; 27.3%) followed by genital herpes (n=85/540; 15.7%), syphilis (n=71/555; 12.8%), gonorrhea (n=14/136; 10.3%), and soft chancre (n=2/10; 20.0%). No episode of urethritis, chlamydia, or trichomoniasis was managed with the recommended antibiotic, dose, and treatment duration. Tables 1, 2, and 3 show the main drugs used for the treatment of urethral syndrome, ulcerative syndrome, and genital warts.

### Comorbidities

A total of 68.1% (n=2,151) of patients had some chronic pathology. Cardiovascular diseases were the most frequent (n=1,382; 43.8%) followed by endocrine (n=845; 26.8%) and digestive disorders (n=541; 17.1%).

**Table 1:** Pharmacological management of episodes of ulcerative syndrome in 3,158 patients with sexually transmitted infections, Colombia

Syphilis (N=555)	n	%
Medications included in the management guide	361	65.0
Benzathine penicillin G	337	60.7
Doxycycline	24	4.3
Other antimicrobial management guide	92	16.6
No antimicrobial handling	102	18.4
Genital herpes (N=540)	n	%
Medications included in the management guide	443	82.0
Acyclovir	414	76.7
Valaciclovir	29	5.4
Other antimicrobial management guide	37	6.9
No antimicrobial handling	60	11.1
Soft chancre (N=10)	n	%
Medications included in the management guide	6	60.0
Azithromycin	3	30.0
Erythromycin	2	20.0
Ceftriaxone	1	10.0
Other antimicrobial management guide	4	40.0
No antimicrobial handling	0	0.0

**Table 2:** Pharmacological management guide of episodes of urethral syndrome in 3,158 patients with sexually transmitted infections, Colombia

Urethritis (N=912)	n	%
Medications included in the management guide	47	5.2
Ceftriaxone + doxycycline	44	4.8
Ceftriaxone + azithromycin	3	0.3
Other antimicrobial management guide	766	84.0
Doxycycline	298	32.7
Ciprofloxacin	185	20.3
Nitrofurantoin	50	5.5
Ceftriaxone	39	4.3
Norfloxacin	28	3.1
Azithromycin	21	2.3
Others	145	15.9
No antimicrobial handling	99	10.9
Gonorrhea (N=136)	n	%
Medications included in the management guide	72	52.9
Doxycycline	41	30.1
Ceftriaxone	31	22.8
Other antimicrobial management guide	51	37.5
Ciprofloxacin	16	11.8
Others	35	25.7
No antimicrobial handling	13	9.6
Chlamydia (N=32)	n	%
Medications included in the management guide	18	56.2
Doxycycline	17	53.1
Azithromycin	1	3.1
Other antimicrobial management guide	6	18.8
Ceftriaxone + ciprofloxacin	2	6.3
Others	4	12.5
No antimicrobial handling	8	25.0
Trichomoniasis (N=28)	n	%
Medications included in the management guide	20	71.4
Metronidazole	16	57.1
Tinidazole	4	14.3
Other antimicrobial management guide	7	25.0
Fluconazole	3	10.7
Others	4	14.3
No antimicrobial handling	1	3.6

**Table 3:** Pharmacological management guide of episodes of genital warts in 3,158 patients with sexually transmitted infections, Colombia

Genital warts (N=939)	n	%
Medications included in the management guide	314	33.4
Podophyllin	314	33.4
Trichloroacetic acid	0	0.0
Other antimicrobial management guide	429	45.7
Silver sulfadiazine	154	16.4
Clotrimazole	93	9.9
Metronidazole	61	6.5
Others	121	12.9
No antimicrobial handling	196	20.9

### Multivariate analysis

The binary logistic regression analysis showed that being a man under 30 years of age, being treated in municipalities other than capital cities, and having received inadequate treatment for the first sexually transmitted infections episode, were associated with a higher probability of another episode during the follow-up period. No variable was protective against this risk (table 4).

**Table 4:** Multivariate analysis of the variables associated with the recurrence of sexually transmitted infections

Variables	Sig	OR	95%CI	
			Lower	Upper
Age <30 years	<0,001	1.729	1.403	2.132
Man	0.007	1.327	1.080	1.631
Treatment in municipalities	0.018	1.439	1.064	1.947
Endocrine diseases	0.077	0.811	0.643	1.023
Having received inadequate treatment in the first episode	<0,001	1.913	1.528	2.395
Condom dispensing	0.187	1.167	0.928	1.467

Sig: statistical significance; OR: odds ratio; CI95%: confidence interval 95%

## Discussion

The main sexually transmitted diseases frequencies, their pharmacological treatment in compliance with the country’s clinical practice guidelines, and the factors associated with having two or more episodes were identified in a cohort of patients from both sexes aged 14 and older in a Colombian population.

Similar to our report, in a study in Spain, 27.1% of the patients had urethral syndrome ^11^, and the most frequently found sexually transmitted infection was genital warts in agreement with the rate reported by Jacob, *et al*. in Germany (51.2%) ^12^ but differing from that found in Belgium, where the most common sexually transmitted infection was chlamydial infection (43.1%) ^13^. According to WHO reports ^4^^,^^6^ and to a prevalence study from Colombia ^7^, infections caused by *Chlamydia trachomatis* are the most common infections in both men and women. However, in our study, chlamydial infections were found in less than 1% of cases due perhaps to the fact that patients were classified according to the ICD-10 codes and many of the sexually transmitted infections classified as unspecified urethritis were likely chlamydial infections.

Only 13.6% of the urethral syndrome, ulcerative syndrome, and genital warts cases treatment adhered to the clinical practice guidelines on antibiotic choice, dose, and duration. Inadequate treatment, including the use of incorrect antibiotics or sub-therapeutic doses, can lead to multiple treatments for the same infection generating an excessive burden on the health system, promoting the development of resistance to antibiotics, and reducing the effectiveness of current treatments ^9^.

The information on this worrisome problem is limited. However, in England, Wetten, *et al*. found that between 2000 and 2011, the proportion of patients treated for chlamydial infections increased from 59.5 to 78.4% and that more than 90% of them were prescribed the recommended antibiotics; in turn, 32.7-53.6% of patients diagnosed with gonorrhea received antibiotic treatment but, although ciprofloxacin was discontinued as the recommended therapy in 2005, the authors found that it was prescribed in 42% of the cases in 2007 and in 20% in 2011 ^14^. In New Zealand, 65% of patients with gonorrhea episodes were managed in compliance with the country guidelines and the adherence was greater in patients treated at sexual health clinics (89%) than at general medicine clinics (52%) ^15^. From 2011 to 2014, 50-52% of patients with gonorrhea in England were managed according to the guidelines ^16^. In contrast, in the Netherlands, 93.3% of patients with gonorrhea were treated with ceftriaxone in compliance with the practice guidelines ^17^ while in Estonia, 48.6% of treatments for gonorrhea did not comply with the clinical practice guidelines and 3.8% were noncompliant for chlamydia ^9^.

Recurrence is among the problems related to inadequate sexually transmitted infections pharmacological management ^3^^,^^9^. In Belgium, reinfections occurred in 15.4% of patients ^13^; in Brazil in 13.6% ^18^; in Canada in 6.4% ^19^, and in our study in 17.6% and we documented a 91% increase in the risk of recurrence when the recommended medication was not administered or when no antimicrobial treatment was indicated. In the United States, Amiri, *et al*. determined that the factors associated with not receiving treatment were being a woman (OR=1.25; 95%CI 1.05-1.50) and residing in a small town (OR=1.49; 95%CI 1.20-1.86) ^20^.

In general, sexually transmitted infections are found more frequently in men as reported in Belgium (57.3%) (13), but contrary to other studies where they predominated in women ^21^^-^^23^. According to several studies, being a man is a risk factor for sexually transmitted infections recurrences as informed in Brazil (OR=4.28; 95%CI 1.31-14.0) ^18^, England (OR=9.9; 95%CI 1.3273.78) ^24^, Spain (HR=1.9; 95%CI 1.3-2.8) ^25^, and in our study (OR=1.32; 95%CI 1.08-1.63). In addition, those under 25 are considered a high-risk population for sexually transmitted infections ^8^^,^^20^ given that according to incidence estimates in the US, about 50% of infections occur in women and men aged 15-24 years ^22^. In Spain, López, *et al*. found that being less than 20 years (HR=4.1; 95%CI 2.1-8.0) was a risk factor for chlamydial reinfections ^25^, which agrees with our findings that those under 30 years had a 72% risk of having a new sexually transmitted infection episode. Older people are more aware of the importance of reproductive health care and of prevention, detection, and treatment of these infections ^20^, so they tend to have stable relationships and use condoms more frequently ^8^.

The use of condoms is one of the main strategies to reduce sexually transmitted infection transmission, but a reduction in their use has been documented among the younger population ^8^. We found that one-fifth of the patients in our study were dispensed condoms in contrast with the case of Brazil, where 76.2% of people reported their use, although only 9.1% used them regularly ^18^. In Almeida, *et al*.’s ^18^ and our study, condom use was not a protective factor against recurrence, maybe because of its inappropriate and/or inconsistent use ^8^.

Our results should be cautiously interpreted since we did not have access to the medical records to confirm sexually transmitted infections etiological or clinical diagnoses. Additionally, we had no data on the education level, the number of sexual partners, the use of illicit drugs, or on antimicrobials, contraceptives, or condoms prescribed outside the health system or not delivered by the dispensing company, nor on the antiviral management received by HIV and hepatitis patients. Finally, it was not possible to establish whether the recurrence was due to the lack of treatment of the sexual partner or partners or to a de novo infection.

We concluded that the majority of sexually transmitted infection patients in our study were not treated in compliance with the Colombian clinical practice guidelines and that those not receiving adequate management had a higher risk of recurrence, as well as men, adolescents, and young adults. For the comprehensive management of sexually transmitted infection patients, prescribing physicians should appropriately select the antibiotic, dose, and duration of treatment to avoid recurrence. The prescription should be based on the available clinical practice guide. The continuous and autonomous updating of medical professionals is very important and health administrators can also play a relevant role in promoting the updating of their prescribers. On the other hand, more studies on antibiotic sensitivity and resistance patterns are required as they are essential for proposing updates in the country’s clinical practice guidelines.
